# 

**Published:** 1885-11-20

**Authors:** 


### ☞ TO OUR SUBSCRIBERS. ☜

Please settle all back dues to the RECORD, and let us commence the New Year with a clear conscience and renewed energy. Friends ! don't forget this, but act promptly. Read next item.

*RECEIPTED.*

For 1884—R. F. McConnell, to April; G. W. Earle, to August.
For 1885—I. T. Babbett, to April; I. L. Hamilton, J. E. Pope, J. R. Reeve, J. C. Beauchamp, W. H. Peek.
1886—T. L. Quillian, Morgan Stokes, Wm. Grymes, Jas. Johnston, Thos. Gaines, I. I. Mallard.

